# Phenolic Acids (Gallic and Tannic Acids) Modulate Antioxidant Status and Cisplatin Induced Nephrotoxicity in Rats

**DOI:** 10.1155/2014/984709

**Published:** 2014-08-11

**Authors:** Seun F. Akomolafe, Ayodele J. Akinyemi, Scholarstical O. Anadozie

**Affiliations:** ^1^Department of Biochemistry, Ekiti State University, P.M.B 5363, Ado Ekiti, Nigeria; ^2^Department of Biochemistry, Afe Babalola University, P.M.B 5454, Ado Ekiti, Nigeria

## Abstract

Cisplatin (*cis*-diamminedichloroplatinum (II) or CDDP), used in the treatment of many solid-tissue cancers, has its chief side-effect in nephrotoxicity. Hence, this study sought to investigate and compare the protective effect of gallic acid (GA) and tannic acid (TA) against cisplatin induced nephrotoxicity in rats. The rats were given a prophylactic treatment of GA and TA orally at a dose of 20 and 40 mg/kg body weight for 7 consecutive days before the administration of a single intraperitoneal (i.p.) injection of cisplatin (CP) at 7.5 mg/kg bwt. The protective effects of both GA and TA on CP induced nephrotoxicity were investigated by assaying renal function, oxidative stress biomarkers, and histopathological examination of kidney architecture. A single dose of cisplatin (7.5 mg/kg bwt) injected i.p. caused a significant increase in some biomarkers of renal function (creatinine, uric acid, and urea levels), with a marked elevation in malondialdehyde (MDA) content accompanied by a significant (*P* < 0.05) decrease in reduced glutathione (GSH) content (103.27%) of kidney tissue as compared to control group. Furthermore, a significant (*P* < 0.05) reduction in kidney antioxidant enzymes (SOD, catalase, GPx, and GST) activity was observed. However, pretreatment with oral administration of tannic acid and gallic acid at a dose of 20 and 40 mg/kg body weight, respectively, for 7 days prior to cisplatin administration reduced histological renal damage and suppressed the generation of ROS, lipid peroxidation, and oxidative stress in kidney tissues. These results indicate that both gallic and tannic acids could serve as a preventive strategy against cisplatin induced nephrotoxicity.

## 1. Introduction

The use of chemotherapy in the treatment of cancer has opened new possibilities for improvement of the quality of life of cancer patients. Despite its success, treatment with some of the most effective anticancer drugs shows a number of symptoms of direct toxicity [1]. In recent years, the mechanism of cisplatin (*cis*-diamminedichloroplatinum (II) or CDDP) induced nephrotoxicity has gradually been elucidated [2]. Studies have shown an increase in lipid peroxides in the renal tissue of CDDP-administered animals [3], a decrease in reduced glutathione levels [1], and the induction of metallothionein [4], an antioxidant. These changes have been considered to result from the generation of reactive oxygen species (ROS). Studies using chemiluminescence or electron spin resonance (ESR) have shown that CDDP generates OH radical [5, 6].

Nephrotoxicity involves kidney damage or dysfunction arising from direct or indirect exposure to drugs and industrial or environmental chemicals. Cisplatin ((*cis*-diamminedichloroplatinum (II) or CDDP)), an anti-neoplastic drug have been reported to induce nephrotoxicity [7]. The kidney which is the major route of cisplatin excretion also accumulates it to a greater degree than other organs [6, 8]. Oxidative stress, inflammation, and apoptosis are some of the mechanisms already established to explain cisplatin induced acute kidney injury [9]. A number of strategies have been proposed for the prevention/management of cisplatin induced nephrotoxicity, since there is no specific treatment, with the use of some synthetic drugs which have been popular. However, these drugs have some associated risks and side-effects [9], hence, the need for natural alternatives of plant origin (plant foods/extracts) with little or no side-effect.

Plants have limitless ability to synthesize aromatic substances such as polyphenols, mainly flavonoids, and phenolic acids, which exhibit antioxidant properties due to their hydrogen-donating and metal-chelating capacities. Polyphenols are secondary metabolites of plants and are widely distributed in plant-derived foods, such as cereals, legumes, nuts, vegetables, and fruits, and in beverages such as green or black tea, and fruit juice. Several hundreds of different polyphenols have been identified in foods [10].

Tannic and gallic acids are two commonly phenolic acids that are structurally related. Tannic acid, a naturally occurring plant polyphenol, is composed of a central glucose molecule derivatized at its hydroxyl groups with one or more galloyl residues, whereas gallic acid is a trihydroxybenzoic acid, also known as 3,4,5-trihydroxybenzoic acid, which is widely distributed in green tea, red wine and grapes, witch hazel, sumac, oak bark, and other plants [11]. Considerable amounts of experimental data on the antioxidant activity of both tannic acid and gallic acids with emphasis on structure-function antioxidant activity have been reported [12]. Also, several authors have demonstrated that tannic acid and other polyphenols have antimutagenic and anticarcinogenic activities [13–16]. Extensive studies have been carried out on the protective effect of cotreatment and posttreatment of phenolic acids against cisplatin induced nephrotoxicity [17]. Hence, this study was carried out to investigate and compare the protective effect of administration of both tannic and gallic acids on normal and cisplatin induced nephrotoxicity in rats.

## 2. Materials and Methods

### 2.1. Experimental Animals

Male albino rats weighing 110–185 g used for this experiment were purchased from a private animal colony, Ikere-Ekiti metropolis. The rats were maintained at 25°C on a 12 hour light/dark cycle with free access to food and water. They were acclimatized under these conditions for two weeks prior to the commencement of the experiments. The experimental study was approved by the Institutional Animal Ethical Committee of the University of Ado-Ekiti, Nigeria.

### 2.2. Chemicals and Reagents

Chemicals such as tannic acid, gallic acid, Oxidized and reduced glutathione, hydrogen peroxide (H_2_O_2_), dithionitrobenzene (DTNB), thiobarbituric acid (TBA), and adrenaline were purchased from Sigma Chemical Co. (St. Louis, MO, USA). Ethanol, acetic acid, H_2_SO_4_, sodium carbonate, sodium citrate, sodium azide (NaN_3_), sodium chloride, potassium dichromate, Tris-HCl buffer, sodium dodecyl sulphate (SDS), and Ascorbic acid were sourced from BDH Chemicals Ltd. (Poole, England). Pharmaceutical grade cisplatin (CP) under the brand name “Cytoplatin 50” was purchased from Cipla Ltd., India. May-Grünwald, Giemsa, and hematoxylin and eosin (H&E) stains were purchased from Hi-Media Labs, Mumbai. All the kits used for bioassay were sourced from RANDOX Laboratories Ltd., Crumlin, County Antrim, UK. Except stated otherwise, all other chemicals and reagents were of analytical grades and the water was glass-distilled.

### 2.3. Study Design and Treatment

After two (2) weeks of acclimatization, 80 male rats were randomly divided into eight (8) groups of ten animals each: Group I served as a normal control and received saline (0.85 w/v%) orally for 7 consecutive days and on the 7th day, 1 hr after receiving the oral saline dose, the rats received a single i.p. injection of saline (0.85%). Group II served as toxicant group and received saline (0.85%) orally for 7 consecutive days. GA was orally administered at two doses, 20 and 40 mg/kg body weight (bwt), to Groups III and IV, respectively, for 7 consecutive days. Also, TA was orally administered at two doses, 20 and 40 mg/kg body weight (bwt), to Groups VI and VII, respectively, for 7 consecutive days. On the 7th day of pretreatment, a single i.p. dose of cisplatin (7.5 mg/kg bwt) after oral treatment of GA and TA was given to the animals in Groups II, III, IV, VI, and VII. Groups V and VIII received only higher dose (40 mg/kg) of GA and TA orally for 7 consecutive days, respectively, and on the 7th day 1 hr GA and TA treatment received a single i.p. injection of saline (0.85%) to ensure that Groups V and VIII received only the higher dose of GA and TA; this was done in order to test that the higher dose did not produce any kind of toxic effects. All the animals were killed after 24 hr of intoxication with cisplatin. The time of killing was based on the preliminary studies. The doses of TA and GA used were actually selected on the basis of preliminary dose escalation studies to determine the minimum dose of TA and GA required to produce an observable effect (data not shown).

### 2.4. Sample Preparation

All the animals were killed after 24 hr of intoxication with cisplatin. The animals were decapitated after an overnight-fast by cervical dislocation. The blood was rapidly collected by direct heart puncture and the plasma was prepared. Uric acid, urea, and creatinine were determined using commercially available kits (Randox Laboratories UK). Arginase activity was determined as described by Kaysen and Strecker [18]. Tissue malondialdehyde (MDA) content was determined as described by Ohkawa et al. [19]. Tissue antioxidant parameters were also determined; superoxide dismutase (SOD) by the method of Alia et al. [20], catalase (CAT) by the method of Sinha [21], reduced glutathione (GSH) by the method of Ellman [22], and glutathione peroxidase (GPx) by the method of Rotruck et al. [23].

### 2.5. Preparation of Plasma

At the end of the experiment, whole blood of the sacrificed rats were collected into EDTA bottles and centrifuged at 800 ×g for 10 min to separate the plasma. The plasma was then decanted into plain sample bottle and stored in a refrigerator prior to analysis.

### 2.6. Preparation of Tissue Homogenates

The rat's tissues (kidney) were rapidly isolated, placed on ice, and weighed. The tissue was rinsed in cold (0.9% w/v) normal saline (1 : 3, w/v) and subsequently homogenized in sodium phosphate buffer (pH 7.4) with (1 : 5 bt w/v) using mortar and pestle as homogenizer and the homogenates were centrifuged at 4,000 ×g. The clear supernatants obtained were used for various biochemical assays [24].

### 2.7. Determination of Plasma Uric Acid Concentration

The uric acid concentration was determined using colorimetric method as described by Collin and Diehl [25] and Morin and Prox [26]. Briefly, 20 *μ*L of distilled water was added to 20 *μ*L of the sample which was mixed with 1 mL of Hepes reagent (50 mM phosphate buffer, 4 mM 3,5-chloro-2-hydroxybenzenesulfonic acid) and enzyme reagent (0.25 mM 4-aminophenazone, peroxidise, and uricase). Thereafter, the mixture was incubated for 5 min at 37°C and the absorbance at 520 nm was taken against reagent blank within 30 min. The uric acid concentration was subsequently calculated against the standard.

### 2.8. Determination of Plasma Urea Concentration

The urea concentration was determined using colorimetric method as described by Searcy et al. [27]. Briefly, 10 *μ*L of sample was added to 0.1 mL of sodium nitroprusside—urease reagent (116 mM EDTA, 6 mM sodium nitroprusside, 1 g/L urease) after which the mixture was incubated for 10 min at 37°C. 2.5 mL of 120 mM diluted phenol and 2.5 mL of 27 mM sodium hypochlorite solution containing 0.14 N sodium hydroxide which was then added to the reaction mixture. Thereafter, the mixture was incubated for 15 min at 37°C and the absorbance at 546 nm was taken against reagent blank within 8 hours. The urea concentration was subsequently calculated against the standard.

### 2.9. Determination of Plasma Creatinine Concentration

The creatinine concentration was determined using colorimetric alkaline picrate method as described by Jaffe method [28]. Briefly, 50 *μ*L of distilled water was added to 2 mL of working reagent (35 mM picric acid and 0.32 M sodium hydroxide) before 50 *μ*L of sample was added. Thereafter, the mixture was allowed to stay for 30 seconds before taking absorbance. The absorbance at 492 nm was taken twice, firstly after 30 sec and secondly after 2 min. The creatinine concentration was subsequently calculated against the standard, using change in the sample absorbance (ΔAbsorbance).

### 2.10. Determination of Arginase Activity

Arginase activity was determined by the measurement of urea produced by the reaction of Ehrlich's reagent according to the modified method of Kaysen and Strecker [18]. The reaction mixture contained 1.0 Mm Tris-HCL buffer, 1.0 mM MnCl_2 _(pH 9.5), 0.1 M arginase solution and 500 mL of the enzyme preparation. The mixture was incubated for 10 mins at 37°C. The reaction was terminated by the addition of 2.5 mL Ehrlich reagent (2.0 g of p-dimethylaminobenzaldehyde in 20.0 mL of concentrated hydrochloric acid and made up to 100 mL with distilled water).

### 2.11. Determination of Superoxide Dismutase (SOD) Activity

Superoxide dismutase (SOD) was determined by the method of Alia et al. [20]. 50 *μ*L of supernatant was treated with 1000 *μ*L of 50 mM carbonate buffer (pH 10.2) and 17 *μ*L of adrenaline (0.06 mg/mL). The absorbance was read at 480 nm in spectrophotometer for 2 minutes at 15-second intervals. SOD activity was expressed as UI per 100 g protein (*ε*_480_ = 4.02 mM^-1 ^cm^−1^).

### 2.12. Determination of Catalase (CAT) Activity

The activity of catalase (CAT) was determined by the method of Sinha [21]. The reaction mixture (1.5 mL) contained 1.0 mL of 0.01 M phosphate buffer (pH 7.0), 0.1 mL of tissue homogenate, and 0.4 mL of 2 M H_2_O_2_. The reaction was stopped by the addition of 2.0 mL of dichromate-acetic acid reagent (5% potassium dichromate and glacial acetic acid were mixed in 1 : 3 ratio). Then, the absorbance was read at 620 nm: CAT activity was expressed as moles of H_2_O_2_ consumed/min/g protein.

### 2.13. Determination of Plasma Reduced Glutathione (GSH) Content

Reduced glutathione (GSH) was determined by the method of Ellman [22]. 1 mL of supernatant was treated with 500 *μ*L of Ellman's reagent (19.8 mg of 5,5′dithiobisnitrobenzoic acid in 100 mL of 0.1% sodium citrate) and 3.0 mL of 0.2 M phosphate buffer (pH 8.0). The absorbance was read at 412 nm in spectrophotometer.

### 2.14. Determination of GPx Activity

The activity of glutathione peroxidase (GPx) was assayed by the method of Rotruck et al. [23]. The reaction mixture containing 0.2 mL of EDTA (0.8 mM, Ph 7.0), 0.4 mL of phosphate buffer (10 mM), and 0.2 mL of tissue homogenate was incubated with 0.1 M of H_2_O_2_ and 0.2 mL of glutathione for 10 min. Oxidation of glutathione by the enzyme was measured spectrophotometrically at 420 nm. The activity of GPx was expressed as l *μ*mol glutathione oxidized/min/g protein.

### 2.15. Determination of Tissue Lipid Peroxidation

The lipid peroxidation assay was carried out using the modified method of Ohkawa et al. [19]. Briefly, 300 *μ*L of tissue homogenate, 300 *μ*L of 8.1% SDS (sodium dodecyl sulphate), 500 *μ*L of acetic acid/HCl (pH = 3.4), and TBA (thiobarbituric acid) were added, and the mixture was incubated at 100°C for 1 hr. Thereafter, the thiobarbituric acid reactive species (TBARS) produced was measured at 532 nm and calculated as malondialdehyde (MDA) equivalent.

### 2.16. Data Analysis

The results of replicate readings were pooled and expressed as mean ± standard deviation. One-way analysis of variance was used to analyze the results and Duncan multiple tests were used for the post hoc analysis [29]. Statistical package for Social Science (SPSS) 10.0 for Windows was used for the analysis. The IC_50_ was calculated using nonlinear regression analysis.

## 3. Results

As evident from Table 1, administration of a single dose of cisplatin (7.5 mg/kg bwt) caused a significant increase in the biomarkers of renal function (creatinine, urea, and uric acid) when compared with the control group that received only saline (0.85% w/v). However, pretreatment with gallic and tannic acids orally at two doses, 20 and 40 mg/kg body weight, respectively, shows a significant (*P* < 0.05) improvement of renal function due to a decrease in the creatinine, urea, and uric acid levels when compared with the induced group (Group 2).

**Figure 1:**
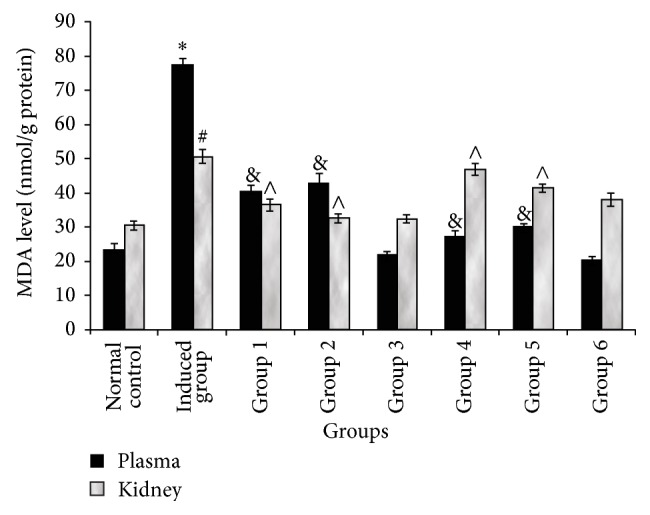
Effect of gallic and tannic acids on kidney and plasma MDA contents in normal and cisplatin induced nephrotoxic rats. 1: Normal control; 2: Induced control, 3: 20 mg gallic acid + cisplatin, 4: 40 mg gallic acid + cisplatin, 5: 40 mg gallic acid only, 6: 20 mg tannic acid + cisplatin, 7: 40 mg tannic acid + cisplatin, and 8: 40 mg tannic acid only. ^∗, #^ Significantly different (*P* < 0.05) from the normal control; ^&,∧^Significantly (*P* < 0.05) different from the induced control.

Also, administration of a single i.p dose of cisplatin (7.5 mg/kg bwt) caused a significant decrease in the biomarkers of oxidative stress [superoxide dismutase (SOD), catalase (CAT), glutathione peroxidase (GPx), and reduced glutathione (GSH)] when compared with the control group that received only saline (0.85% w/v) (Table 2). However, pretreatment with gallic and tannic acids orally at two doses, 20 and 40 mg/kg body weight, respectively, shows a significant (*P* < 0.05) improvement in the body's antioxidant status by an increase in the activities of superoxide dismutase (SOD), catalase (CAT), glutathione peroxidase (GPx), and reduced glutathione (GSH) when compared with the induced group (Table 2). Likewise, there was a significant (*P* < 0.05) increase in the malondialdehyde (MDA) content in rat kidney administered a single i.p dose of cisplatin (7.5 mg/kg bwt). However, both pretreatment with gallic and tannic acids orally at two doses, 20 and 40 mg/kg body weight, respectively, shows a significant (*P* < 0.05) reduction of MDA content in rat kidney when compared with the induced group (Figure 1).

**Figure 2:**
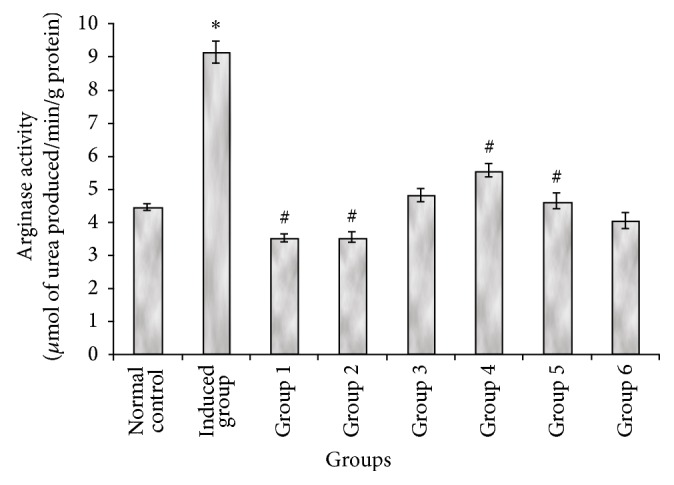
Effect of gallic and tannic acids on arginase activity in the kidney of normal and cisplatin induced nephrotoxic rats. 1: Normal control; 2: Induced control, 3: 20 mg gallic acid + cisplatin, 4: 40 mg gallic acid + cisplatin, 5: 40 mg gallic acid only, 6: 20 mg tannic acid + cisplatin, 7: 40 mg tannic acid + cisplatin, and 8: 40 mg tannic acid only. ^*^ Significantly (*P* < 0.05) different from the normal control; ^#^Significantly (*P* < 0.05) different from the induced control.

Figure 2 revealed that administration of a single i.p dose of cisplatin (7.5 mg/kg bwt) caused a significant (*P* < 0.05) increase in arginase activity when compared with the control group that received only saline (0.85% w/v). However, pretreatment with gallic and tannic acids orally at two doses, 20 and 40 mg/kg body weight, respectively, shows a significant (*P* < 0.05) decrease in arginase activity when compared with the induced group (Group 2).

**Figure 3:**
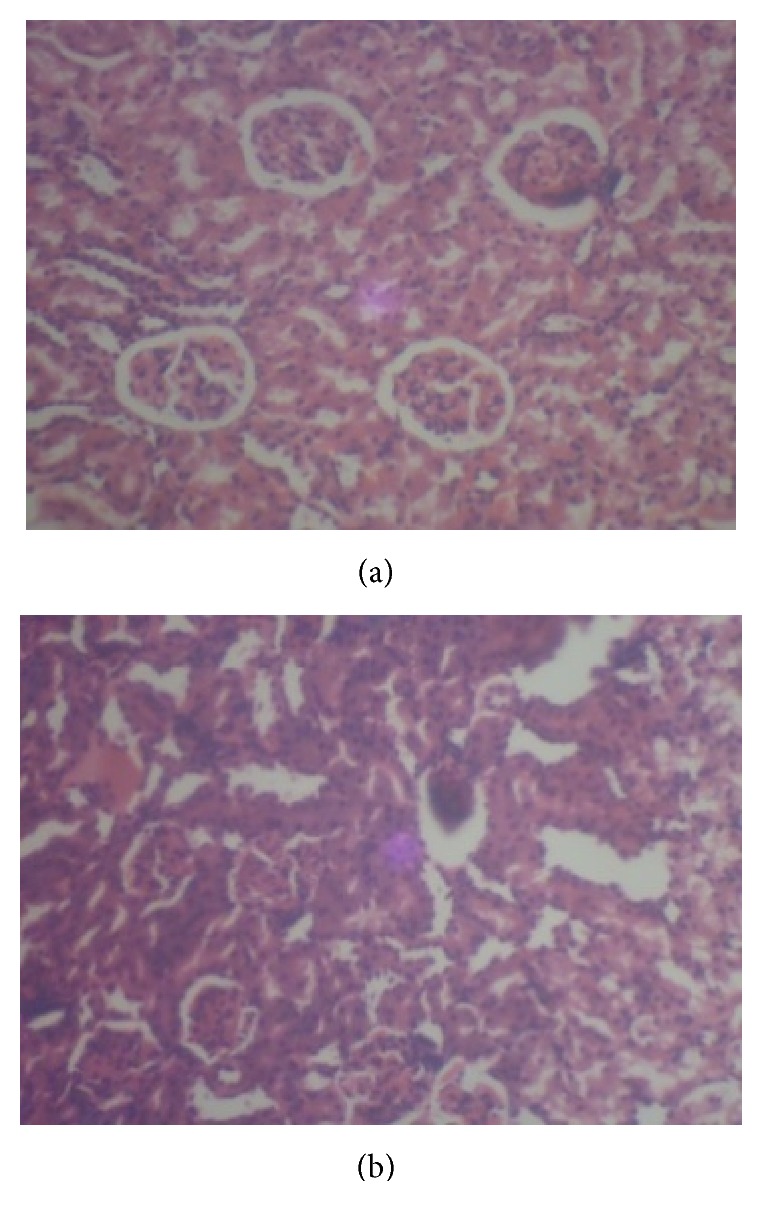
Histopathological views (×400) of the kidney showing severe and generalized tubular epithelial cell necrosis associated with diffuse tubular lumina in rat administered a single i.p dose of cisplatin (7.5 mg/kg bwt) (b), while no damage is noticed in normal control rats (a).

**Figure 4:**
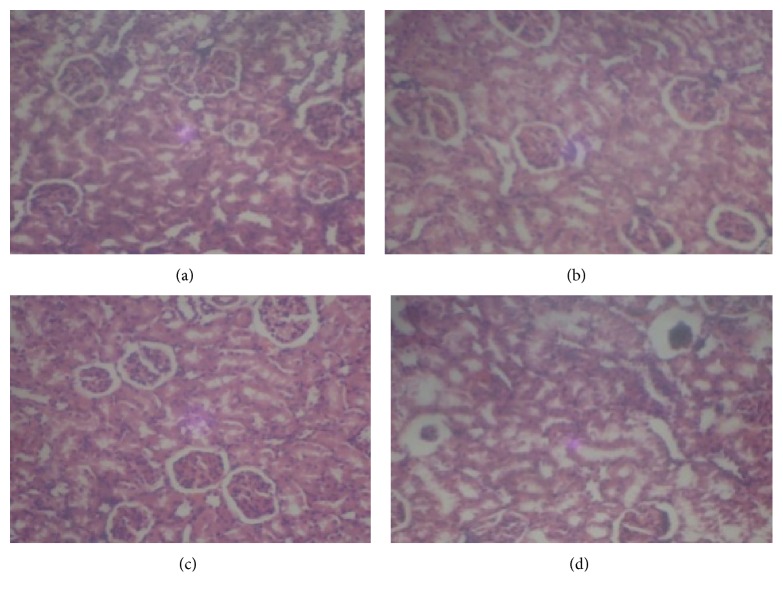
Histopathological views (×400) of the kidney showing a marked improvement on kidney damage in rats pretreated with gallic and tannic acids. ((a) and (b)) gallic acid (20 and 40 mg/kg bwt) + cisplatin and ((c) and (d)) tannic acid (20 and 40 mg/kg bwt) + cisplatin.

**Figure 5:**
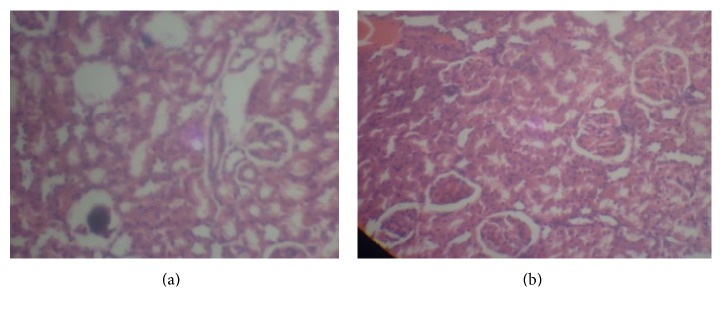
Histopathological views (×400) of the kidney showing no damage in rats pretreated with gallic acid (40 mg/kg bwt) (a) and tannic acid (40 mg/kg bwt) (b) only without cisplatin.

Photomicrographs of kidney sections from various treatment groups are shown in Figures 3, 4, and 5. Histopathological examination of sections from rat kidney administered a single i.p dose of cisplatin (7.5 mg/kg bwt) shows severe and generalized tubular epithelial cell necrosis associated with diffuse tubular lumina when compared with the control without cisplatin. However, pretreatment with gallic and tannic acids orally at two doses, 20 and 40 mg/kg body weight, respectively, shows a marked improvement on kidney damage.

## 4. Discussion

About 25% of most commonly used drugs in intensive care units (ICUs) are potentially nephrotoxic and are recognized as considerable health and economic burden worldwide [1]. Among them, cisplatin, when used in cancer chemotherapy, induces renal impairment and acute renal failure by induction of reactive oxygen species, tubulointerstitial inflammation, and apoptosis [7]. Although various studies have been reported on the benefits of several agents in cisplatin induced renal toxicity, the basis of nephroprotection remains elusive [6]. This makes the search for strategies to prevent nephrotoxicity constitute an active area of investigation. Abundant evidences suggest the involvement of oxidative stress in the pathogenesis of cisplatin nephrotoxicity [8]. Hence, it is reasonable to assume that a reinforcement of antioxidant defense of renal tissue by exogenous antioxidant such as phenolic acids should be a strategy to protect the kidney from the oxidative damage.

In the present study, we compare the protective effect of administration of gallic and tannic acids (two commonly phenolic acids that are structurally related) of the same dosage against normal and cisplatin induced renal injury in rats for the first time by attenuating renal oxidative stress. The elevations of key kidney function biomarkers such as creatinine, uric acid, and urea have been suggested to be indicative of reduced renal functions [30, 31]. Thus, estimation of plasma creatinine and uric acid has been employed as key test to assess kidney function [30, 31].

In the present study, the observed elevation in plasma creatinine, urea, and uric acid levels in induced rats indicates a reduction in kidney function and hence nephrotoxicity (Table 1). This finding is consistent with that reported by earlier studies [32, 33]. However, the restoration of the plasma creatinine, urea, and uric acid level in rats treated with both gallic and tannic acids (Table 1) suggests that both phenolic acids have the ability to prevent kidney damage and protect the kidney against nephrotoxicity. This, however, may be a function of their antioxidant properties and ability to inhibit arginase activity (Figure 2). Cisplatin increased arginase activity in the rat's kidney, while the pretreatment with both gallic and tannic acids at two doses (20 and 40 mg/kg bwt), respectively, for 7 days resulted in a decrease in kidney arginase activity (Figure 2).

Arginase is a hydrolytic enzyme responsible for the conversion of L-arginine to L-ornithine and urea. Ornithine is an important biosynthetic precursor of polyamines which have been implicated to facilitate cell proliferation in certain cancer cells [34]. Another important enzyme, endothelium nitric oxide synthase (eNOS), competes with arginase for the same substrate, arginine. eNOS is involved in the production of nitric oxide (NO) from arginine. The NO produced plays an important role in both regulating renal hemodynamics and modulating inflammatory and proliferating response to various stimuli. Therefore, inhibition of arginase activity slows the progression of renal failure in renal ablation.

The increase in the kidney and plasma MDA content (Figure 1) in the induced rats suggests lipid peroxidation. This agreed with earlier studies where administration of cisplatin caused inflammation and lipid peroxidation [6, 7]. This could be as a result of increased hydrogen peroxide concentration produced in the kidney due to the depletion of antioxidant enzymes: superoxide dismutase (SOD), reduced glutathione (GSH), and catalase (CAT) activity (Table 2). This also is consistent with earlier study where depletion of SOD, CAT, and GPx in rats resulted in increased MDA concentration due to lipid peroxidation [35]. The depletion of these antioxidants suggests that cisplatin induced nephrotoxicity could be a result of oxidative stress or suppression of the antioxidant enzymes, as previously reported by earlier studies [35–37]. However, the reduced kidney and plasma MDA content (Figure 1) and restoration of SOD, GSH, and CAT activities (Table 2) in pretreated rats suggest an improvement in the in vivo antioxidant status, which may be a function of the antioxidant properties of the phenolic acids (gallic and tannic acids).

Reduced glutathione (GSH) has a multiple role as an antioxidant agent. It functions as a scavenger of ROS, including hydroxyl radicals and singlet oxygen [38]. Therefore, the observed decrease in the kidney GSH level (Table 2) in the induced rats suggests cisplatin induced nephrotoxicity, which is associated with a drastic reduction in kidney GSH content. This finding is consistent with earlier studies where GSH depletion was suggested to be due to the interaction of cisplatin with the molecules contain sulfhydryl groups [39, 40]. However, restoration of GSH levels in the pretreated rats suggests the antioxidant and nephroprotective properties of the phenolic acids (Table 2).

Furthermore, histopathology study revealed normal glomerulus and tubules with intact renal architecture in normal (Figure 3(a)), gallic (Figure 5(a)), and tannic acid (Figure 5(b)) group without cisplatin injection. Degenerated tubular structures with vacuolization and loss of architecture were seen in cisplatin induced group (Figure 3(b)). Pretreatment with both gallic and tannic acids at two doses (20 and 40 mg/kg bwt), respectively, for 7 days resulted in excellent protection against nephrotoxicity induced by cisplatin and showed predominant normal kidney morphology (Figures 4(a), 4(b), 4(c), and 4(d)).

## 5. Conclusion

The results of the present study revealed that oxidative stress and apoptosis/necrosis play an important role in pathogenesis of cisplatin nephrotoxicity. Gallic and tannic acids, two important pharmacologically active phenolic compounds, reduced cisplatin induced functional and histological renal damage. Furthermore, they suppressed the generation of ROS, lipid peroxidation, and oxidative stress in kidney tissues. These results indicated that both gallic and tannic acids exhibit nephroprotective effect and the possible mechanism of action by which they exert this effect could be due to their antioxidant properties and inhibition of arginase activity. However, tannic acid exhibited better nephroprotective potential than gallic acid which may be due to the glycosidation with a glucose moiety.

## Figures and Tables

**Table 1:** Effect of tannic and gallic acids on renal function test in normal and cisplatin induced nephrotoxic rats.

Groups	Treatments	Creatinine (mg/dl)	Urea (mg/dl)	U/A (mg/dl)
1	Normal control	44.04 ± 1.2	6.82 ± 0.8	7.12 ± 1.9
2	Induced control	131.49^b ^± 2.3	8.51^b ^± 0.7	11.09^b ^± 2.2
3	20 mg gallic acid + cisplatin	47.23^c ^± 1.8	7.05^c ^± 1.0	5.81^c ^± 0.8
4	40 mg gallic acid + cisplatin	20.43^c ^± 2.7	6.29^c ^± 1.5	4.96^c ^± 0.9
5	40 mg gallic acid only	16.59 ± 3.1	6.04 ± 1.6	4.80 ± 1.4
6	20 mg tannic acid + cisplatin	67.66^c ^± 2.3	7.31^c ^± 0.5	8.24^c ^± 3.5
7	40 mg tannic acid + cisplatin	11.49^c ^± 1.7	7.04^c ^± 0.5	4.33^c ^± 0.7
8	40 mg tannic acid only	7.66 ± 0.4	6.53 ± 0.4	5.37 ± 1.3

Values represent mean ± standard deviation (*n* = 6). ^b^Significantly (*P* < 0.05) different from the normal control. ^c^Significantly (*P* < 0.05) different from the induced control.

**Table 2:** Effect of tannic acid and gallic acids on antioxidant content level in normal and cisplatin induced nephrotoxicity in rats.

Groups	SOD	CAT	GPx	GSH
1	2.01 ± 0.95	0.18 ± 0.10	0.21 ± 0.03	0.24 ± 0.02
2	1.06^b^ ± 0.53	0.09^b^ ± 0.06	0.13^b^ ± 0.00	0.13^b^ ± 0.05
3	1.31^c^ ± 0.62	0.16^c^ ± 0.03	0.17^c^ ± 0.03	0.17^c^ ± 0.04
4	1.75^c^ ± 1.24	0.16^c^ ± 0.01	0.22^c^ ± 0.05	0.18^c^ ± 0.02
5	2.05 ± 0.73	0.19 ± 0.02	0.23 ± 0.08	0.25 ± 0.02
6	1.97^c^ ± 0.93	0.12^c^ ± 0.03	0.16^c^ ± 0.00	0.17^c^ ± 0.03
7	2.39^c^ ± 0.21	0.17^c^ ± 0.20	0.23^c^ ± 0.11	0.19^c^ ± 0.00
8	4.02 ± 0.95	0.18 ± 0.00	0.25 ± 0.14	0.22 ± 0.03

Values represent mean ± standard deviation (*n* = 6). 1: induced control, 2: normal control, 3: 20 mg gallic acid + cisplatin, 4: 40 mg gallic acid + cisplatin, 5: 40 mg gallic acid only, 6: 20 mg tannic acid + cisplatin, 7: 40 mg tannic acid + cisplatin, and 8: 40 mg tannic acid only.

^b^Significantly (*P* < 0.05) different from the induced control. ^c^Significantly (*P* < 0.05) different from the induced control.
